# Occupational accidents in professional dance with focus on gender differences

**DOI:** 10.1186/1745-6673-8-35

**Published:** 2013-12-17

**Authors:** Eileen M Wanke, Michael Arendt, Helmgard Mill, David A Groneberg

**Affiliations:** 1 Institute of Occupational Medicine, Charité - Universitätsmedizin Berlin, Thielallee 69, 14195 Berlin, Germany; 2 Institute of Occupational Medicine, Social Medicine and Environmental Medicine, Goethe-University, Am Sternkai 7, 60590 Frankfurt am Main, Germany; 3 Unfallkasse Berlin, Department Prevention, Culemeyer Strasse 2, 12277 Berlin, Germany

**Keywords:** Traumatic injury, Dancer, Ballet, Male, Female

## Abstract

**Background:**

Classical dance comprises gender specific movement tasks. There is a lack of studies which investigate work related traumatic injuries in terms of gender specific differences in detail.

**Objective:**

To define gender related differences of occupational accidents.

**Methods:**

Basis for the evaluation were occupational injuries of professional dancers from three (n = 785; f: n = 358, m: n = 427) state theatres.

**Results:**

The incidence rate (0.36 per year) was higher in males (m: 0.45, f: 0.29). There were gender specific differences as to the localizations of injuries, particularly the spine region (m: 17.3%, f: 9.8%, p = 0.05) and ankle joint (m: 23.7%, f: 35.5%, p = 0.003). Compared to male dancers, females sustained more injuries resulting from extrinsic factors. Significant differences could specifically be observed with dance floors (m: 8.8%, f: 15.1%, p = 0.02). There were also significant gender differences observed with movement vocabulary.

**Conclusion:**

The clearly defined gender specific movement activities in classical dance are reflected in occupational accidents sustained. Organisational structures as well as work environment represent a burden likewise to male and female dancers. The presented differences support the development of gender specific injury prevention measures.

## Background

Professional classical dance comprises gender specific work contents. For female dancers, this contains the work with *pointe shoes* and the ‘being lifted’ while working with a (male) partner. Prerequisites for females are an aesthetical ideal characterized by a low body weight, an androgynous appearance and a high flexibility. Work contents of male dancers are different, particularly due to the dynamic movement vocabulary (e.g. very large jumps (*grand allegro*)), ranging into space and width, jump combinations, variations of multiple *pirouettes* (especially with one leg outstretched into space as well as jumped turns (*tour en l`air*).

Since a healthy body is the basis to maintain a dance position in a professional dance company, any physical restriction (e.g. caused by an occupational accident) can threaten a dancer’s career [1]. Hence, specific preventive measures are of great significance [2-5]. Although there have been several investigations on injuries in professional dancers, investigations comparing occupational accidents in classical dance with focus on gender related differences are still lacking. Of particular interest is whether the gender specific movement vocabulary in training, rehearsals and performances is reflected in the traumatic injuries sustained by dancers. Therefore, it is the aim of this study to evaluate health hazards resulting from traumatic injuries (modified after Bronner et al. [6] in professional ballet dancers with regard to gender specific differences. The findings may stimulate the development of future studies such as qualitative analyses and/or longitudinal evaluations regarding gender related issues or gender specific preventative measures.

## Methods

Basis for the quantitative cohort study analysis were occupational accidents in professional classical dancers from Berlin Theaters over a period of 17 years (1995-2011). The aspect that the data used had been collected over a longer period of time as well as qualitative aspects remain unconsidered.

### Definition of a work-related accident

According to the German Social Accident Insurance Institution for the public sector in Berlin [edited for the review process] (UKB), a work accident (= traumatic injury) is defined as “a time-limited incident that mainly acts on the insured person’s body from the outside during work or on the way to work or home resulting in physical damage or death”.

The UKB injury severity codes range from “minor” (time loss [TL] for up to 5 days), to “mid-level” (TL for more than 5 days) to “severe” (TL for more than 6 weeks).

Analyzing the nature of causes of traumatic injuries, intrinsic factors (nutritional and training status, physical prerequisites, technical skills) as well as extrinsic factors (training plan, microclimatic conditions, lighting, partner, props/equipment, costume, floor) can be differentiated [7-9].

The work contents in this study comprise training, rehearsal and performance.

### Data collection und subject groups

All traumatic work-related injuries in dancers from three state theatres (n = 785; f: n = 358, m: n = 427) with a mean age of 28.7 ± 5.3 years (m: 28.5 ± 5.4 yrs; f: 28.9 ± 5.2 yrs) were documented in UKB-standardized work accident reports and exposure data from the UKB. The data originates from classical dance. Professional dancers employed in Germany are legally bound by law to be members of the UKB. Each of the injuries evaluated in this study was examined by a medical doctor (usually an orthopaedic surgeon) authorized to deal with work accidents which are documented by him/her in UKB standardized injury forms (F1000, DAB resp.) soon after the accident occurred, preferably on the day the accident happened. The form provides information regarding the injured person (anonymized for this study), beginning and end of working hours of the day the accident occurred, the date when employment with the theater began, time, cause and circumstances of the accident, medical findings, diagnosis, treatment, and rehabilitation. Registration and administration of the accident reports, generally not accessible to the public, is centralized at the UKB.

### Dance style

In our study classical dancers were considered. **Classical dance** (ballet) is the basis of professional dance. It is clearly defined and subject to a structured terminology [10]. Characteristic features are the turn out of the stretched leg (*en dehors*) and the *pointe* work for female dancers.

### Data analysis

The data were evaluated anonymously. Results were calculated using the PASW Statistics software package, Version 18.0 and Excel 2007. Predominantly, the evaluation was carried out in the form of frequency analyses. Chi-Square Tests were used at crucial points to evaluate the differences between the groups. The significance level was set at α=0.05.

### Limitations of the study

Due to existing law, it is safe to assume that each occupational accident is reported. The total number of more than one occupational accident sustained by one dancer is very low (<2%) and was neglected because it had no influence on the statistics.

The UKB severity codes are valid for all types of occupations and not optimally applicable in dance because even minor injuries may threaten a dancer’s career. Although, as in cases of this sort, the UKB methodology for recording injuries lacks the elegant specificity one would prefer, it serves the needs of this study quite adequately. Unless they were not part of the standardized injury form, dance specific recommendations for the evaluation of injuries could not be considered [6,11-13].

### Medical ethics

Due to the research design, neither permit from the Ethics Committee nor from the Institutional Review Board was required. From a data protection perspective this data does not require any particular protection for they are not personal data.

## Results

A total of 785 occupational accidents was reported for an average of 46.2 ± 3.3 (m: 25.1, f: 21.1) per theatrical season with an incidence rate of 0.36 per year (m: 0.45, f: 0.29). This corresponds to an average of 0.2/1000 work hrs (m: 0.3/1000 work hrs, f: 0.2/1000 work hrs). An annual increase in injuries could not be observed over the course of the study. In relation to the total number of dancers employed at the three theaters, acute injuries were more frequently sustained by males over the course of a theatrical season.

### Localization

**Figure 1:**
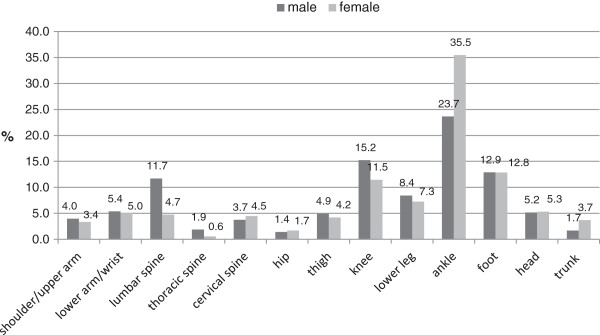
Lokalisation of occupational accidents (n=785).

Gender related differences could be found (p=0.015). The most commonly injured region in either sex was the lower extremity (m: 66.5%, f: 72.9%), followed by the spine (m: 17.3%, f: 9.8%, p=0.05) and the upper extremity in males (9.4%, f: 8.4%), resp., head/trunk in females (8.9%, p=0.83). With the lower extremity, the ankle joint (m: 23.7%, f: 35.5%, p=0.003) was the most frequently injured localization in either sex, followed by the knee joint (m: 15.2%, f: 11.5%), resp., the remaining foot/toes (m: 12.9%, f: 12.8%;). There were visible but non-significant gender specific differences with the upper and lower extremity. Significant gender specific differences were observed in the spine region (m: 17.3%, f: 9.8%) particularly with the more than twice affected lumbar spine in male dancers (LS: m: 11.7%, f: 4.7%; thoracic spine (TS): m: 1.9%, f: 0.6%; cervical spine (CS): m: 3.7%, f: 4.5%) (Figure 1).

### Injured structures and injury types

The injured structures show significant gender-related differences (p<0.02), although joints (particularly ankle joint and knee joint) including ligaments were the most commonly affected structures in either sex (m: 61.0%, f: 64.3%) with muscle injuries (calf and thigh) following in males (22.1%, f: 15.2%) and bone injuries in females (15.7%, m: 12.0%). Sprains, contusions, and pulled muscles belonged to the most common types of injury in either gender (p=0.47) in all investigated fields, with sprains as the most common type in males (29.6%, f: 26.7%) and pulled muscles in females (29.6%, m: 27.9%), followed by contusions in either sex (m: 13.6%, f: 15.2%).

According to the general (non dance-specific) severity codes of the [edited for the review process] UKB ([edited for the review process]), where type of injury, costs and duration of treatment are considered, most injuries in dancers were graded as “minor” and “mid-level” (m: 90.7%, f: 90.6%, p=0.43). The percentage of “severe” injuries (e.g. intra-articular knee injuries, cruciate ligament ruptures) was higher in males (m: 10.3%, f: 9.5).

61.2% (m) and 58.6% (f) of the dancers continued to work after sustaining a work accident; with 38.8% (m) and 41.4% (f) discontinuing (stopped to dance) (p=0.5). There were no significant differences in terms of time loss due to illness. A total of 90% of the male dancers, resp. 85.5% of their female counterparts were more than three day absent from work (p=0.43).

### Time of the injury

The majority of injuries occurred during the first three hours after start of work (m: 72.4%, f: 67.6%). Most injuries were sustained by males during the third hour (m: 26.1%, f: 23.2%), with females in the second hour (f: 23.5%, m: 25.8%) (p=0.45). In terms of time there were also visible non significant differences with traumatic injuries occurring during the day and the theatrical season. The most common injuries occurred in the 4th quarter (m: 33.1%, f: 30.9%), followed by the 2nd (m: 27.7%, f: 28.1%) and the 1st (m: 24.9%, f: 28.0%) (p=0.72). Approximately one third of all injuries sustained during the day occurred by noon (m: 33.1%, f: 32.0%, p=0.61).

Upon closer observation of the occupational accidents subject to the period of time between beginning of employment and occupational accident, significant differences (p=0.02) could be observed. Most occupational accidents in either sex occurred in the second and third year after starting the employment (m: 52.6%, f: 54.8%), with male dancers (21.8%) in the third and females in the first year (18.1%). Female dancers sustained traumatic injuries twice as much as their male counterparts in the first year (Table 1).

**Table 1:** Occupational accidents over the course of time in % (n=785)

	**Male (n=427)**	**Female (n=358)**	**p value**
**A. Course of the year**			p=0.72
1^st^ quarter	24.9	28.1	
2^nd^ quarter	27.7	28.1	
3^rd^ quarter	14.2	12.8	
4^th^ quarter	33.1	30.9	
**B. Period of time from start of work to time of accident**			
0-60 min	20.5	20.6	p=0.45
61 min-120 min	25.8	23.5	
121 min-180 min	26.1	23.2	
>180 min	32.6	32.7	
**C. Injuries during the day**			p=0.61
10.01-14.00	42.9	41.9	
14.01-18.00	16.2	20.9	
18.01-22.00	39.8	35	
Later	1.4	2.2	
**D. Time period between beginning of employment and occupational accident**			
1^st^ year	9.0	18.1	p=0.02
2^nd^ year	27.8	32.9	
3^rd^ year	24.8	21.9	
4^th^ year	21.8	17.4	
Later	16.7	9.7	

### Causes

Table 2 shows the objects causing dance style-related work related accidents. The causes for traumatic injuries differed significantly only in part between males and females. Injuries were most commonly caused by intrinsic factors (m: 64.0%, f: 57.1%, p=0.15), and usually a combination of various factors. The most common extrinsic factors in either sex were the dance partner (m: 17.1%, f: 19.1%, p=0.14) with the dance floor resulting in significantly more traumatic injuries in female dancers (m: 8.8%, f: 15.1%, p=0.02).

**Table 2:** Objects causing accidents in professional dance (n=785)

**Objects causing accidents**	**Male (n=427) n *(%)***	**Female (n=358) n *(%)***	**p value**
Intrinsic* (training)	53 *(12.4)*	35 *(9.7)*	p=0.15
Intrinsic* (rehearsal)	144 *(33.1)*	113 *(31.7)*	
Intrinsic* (performance)	79 *(18.6)*	56 *(57.1)*	
Extrinsic** total	154 *(36.0)*	152 *(15.7)*	
**Comparison of the three most common extrinsic factors**			
Dance floor	38 *(8.8)*	54 (*15.1)*	p=0.02
Dance partner	73 *(17.1)*	67 *(19.1)*	p=0.14
Props/equipment	14 *(3.3)*	13 *(3.7)*	p=0.7
Others	29 *(6.7)*	17 *(4.8)*	p=0.03

*intrinsic factors: e.g. nutritional status, training status, physical prerequisites, technical skills.

**extrinsic factors: e.g. floor, props/equipment, dance partner, costume, training plan, microclimatic conditions, lighting.

### Work contents

Work contents differed only non-significantly between males and females at the time of the occupational accident. The vast number of accidents in either gender occurred during rehearsals (m: 55.6%, f: 52.3%), followed by performances (m: 30.3%, f: 27.3%) and the daily training (m: 11.6%, f: 14.6%).

### Dance activities before the accident happened

**Figure 2:**
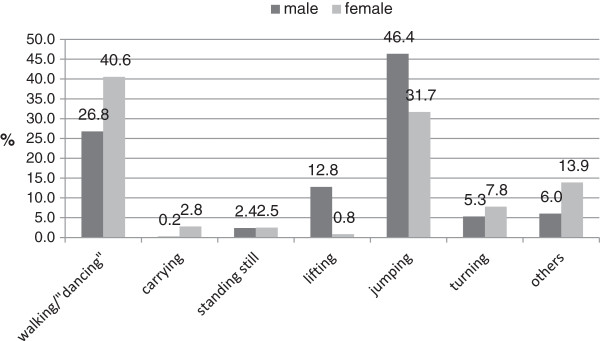
Dance activities before the accident happened in professional dance (n=785).

Highly significant gender differences (p<0.001) (Figure 2) were observed with movement contents where explicitly named jumps (*allegro*, *grand allegro, cabriole, grand jeté*) resulted in the most frequent traumatic injuries in males (sprains, pulled muscles). Female dancers sustained traumatic injuries while performing smaller steps/dance combinations. One of three traumatic injuries in females occurred after jumps, one of five after turns, resp. the landing thereafter. Male dancers sustained traumatic injuries after lifting a partner (12.8%) with only 0.8% of females sustaining a trauma.

### Movements resulting in an accident

**Figure 3:**
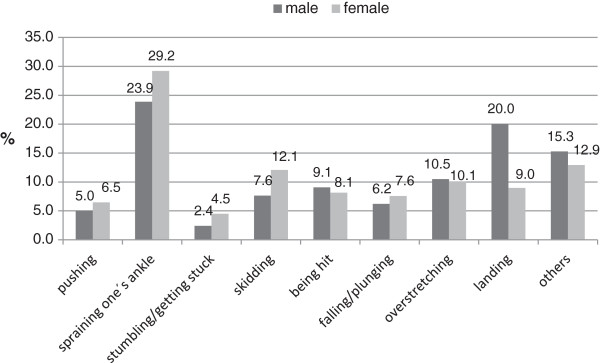
Movements resulting in an occupational accident in professional dance (n=785).

Regarding gender, there were highly significant differences with movements resulting in occupational accidents (p<0.001). The most common movement is “spraining one’s foot” in either sex (m: 23.9%, f: 29.2%), followed by “slipping” in females (12.1%), resp. “landing” in males (12.1%) as well as “overstretching“ in either sex (m: 10.5%, f: 10.1%) (Figure 3).

## Discussion

Professional classical dance comprises gender specific work contents. Even for non-dance experts the differences are less visible during the training but rather obvious in rehearsals and performances. The aim of this study was to analyze and evaluate occupational accidents in terms of gender-related differences. It was of particular interest to find out whether diverse movement contents are reflected in the occupational accidents sustained.

The number of annual traumatic injuries seems surprisingly low. Nevertheless, injuries in one out of two male dancers and one out of three females confirm incidence rates below those of other authors [1-3,8,9,14]. The reason for this is that traumatic and overuse injuries were not analyzed separately in almost all relevant studies which skews a comparison with the results of this study [14-19].

Male dancers appear to be more susceptible to injuries than their female counterparts. A possible reason for this may be the lower number of employed male dancers and the disproportionally heavy workloads combined therewith. Another reason may be the relatively high risk factors inherent in the male movement vocabulary (large jumps, lifts, etc.) [15,16].

There were gender-specific differences in the spine region and ankle joint. As already reported by Orishimo et al. [20] and supported by this study, gender-related differences in the knee joint region often observed in other types of sport do not seem to appear in classical dance. In the spine region, the lumbar spine was affected ca. 2.5 times as much in males as in females which is reflected by the high workloads during jumps (landings) and lifts. This partly aligns with the results of other authors [1,21-23]. Compared to male dancers, cervical spine and ankle joint were the most commonly injured localizations in females, presumably resulting from high mobility, terminal movements performed and aesthetic ideal. Particularly in the ankle joint region, a high mobility combined with the aesthetic ideal (high instep) and *pointe* shoes can increase the instability of joint structures and thus the injury risk. This could explain the high number of ankle joint injuries in female dancers [24,25]. Although the differences between injured structures and types of injuries were not significant, they underline the high injury risk potential implied in big jumps performed by male dancers with not only joint structures including ligaments but also muscular structures affected.

Preventive approaches to reduce traumatic injuries [26-30] could possibly be a complementary coordination- and (dance specific or non dance specific) strength training (e. g. Pilates) as well as the development of a good basic endurance.

Gender differences could hardly be observed in terms of time factors which imply that organisational aspects do not have a gender-specific influence on the occurrence of occupational injuries. Significant differences were merely observed in terms of the time span between taking up employment and occupational accident. In the first year of their employment, females were almost twice as much injured as males. It is still to be clarified/analyzed/evaluated what the reasons are (e.g. higher competitive pressure in female dancers, aesthetic ideal, extrinsic factors etc.) [23].

Intrinsic factors are more common in terms of occupational accidents sustained than extrinsic ones. One reason could be that working tool and dancer’s body are most often identical in classical dance. Extrinsic factors seem to play a more significant role in accidents sustained by female dancers compared to their male counterparts which is documented by the analysis of accident causes. Independent from the fact that intrinsic factors most commonly result in occupational accidents in classical dancers of either sex – as already observed by other authors [9,15] - dance floors seem to be of greater significance to females than to males. This fact is due to the combination dance floor, movement elements and *pointe* shoes that are in many aspects more demanding than the footwear of males. A top-quality dance floor is here all the more important [31-37].

In the gross, these results underline the significance of exogenous factors that could substantially contribute to injury prevention [5,34,38] measures, provided an optimal state/condition of dance floors and costumes is ensured and props/equipment are either dispensed with or offer appropriate safety without affecting/limiting the artistic licence/freedom.

Although work contents resulting in occupational accidents showed only slight differences with either sex, highly significant differences could be observed with the movement elements performed at the time of the accident. This was completely reflected by the gender specific tasks to be performed. Jumps performed by males and smaller – sometimes very fast –dance movements performed by females were the most common causes resulting in occupational accidents. After a supination trauma, the highly specific landing (externally rotated pivot leg) completing a jump resulted in health hazards to male dancers. In females it was but slipping movements resulting in health hazard. This indicates the significance of dance floors in terms of occupational accidents and their significance for injury prevention measures (5,32).

## Conclusions

The clearly defined gender specific movement activities in classical dance are reflected in occupational accidents sustained. Organisational structures as well as work environment represent a burden likewise to male and female dancers. The presented differences support the development of gender specific injury prevention measures.

## Competing interests

No financial support or other donations were received for this investigation.

## Authors’ contributions

EMW has been drafting the manuscript and has made substantial intellectual contributions to the interpretation of data. MA has made substantial contributions to conception and design and acquisition of data. HM has been involved in revising the manuscript critically for important intellectual content. DAG has made substantial contributions to conception and design of this study and has given final approval of the version to be published. All authors read and approved the final manuscript.
